# Bioinformatics Analysis and Functional Characterization of the CFEM Proteins of *Metarhizium anisopliae*

**DOI:** 10.3390/jof8070661

**Published:** 2022-06-24

**Authors:** Ni Cai, Rong Liu, Duozi Yan, Neng Zhang, Kaihui Zhu, Daogang Zhang, Xiangqun Nong, Xiongbing Tu, Zehua Zhang, Guangjun Wang

**Affiliations:** The State Key Laboratory for Biology of Plant Diseases and Insect Pests, Institute of Plant Protection, Chinese Academy of Agricultural Sciences, Beijing 100193, China; 15027173107@163.com (N.C.); liurong20220622@163.com (R.L.); yanduozi@163.com (D.Y.); zhang1998neng@163.com (N.Z.); zkh0.0@hotmail.com (K.Z.); ippleslie04@163.com (D.Z.); zhangzehua@caas.cn (Z.Z.); wangguangjun@caas.cn (G.W.)

**Keywords:** CFEM domain, candidate effector, *Metarhizium anisopliae*, signal peptide, PCD

## Abstract

The entomopathogen *Metarhizium anisopliae* is a facultative rhizosphere or endophytic fungus available for managing pests and improving plant growth. The CFEM (common in fungal extracellular membrane) proteins form a unique group in fungi but are rarely reported in entomopathogens. In this study, we cloned and identified 13 CFEM genes from *M. anisopliae* (MaCFEMs). Sequence alignment and WebLogo analysis showed that eight cysteines were the most conserved amino acids in their CFEM domain. Phylogenic analysis suggested that these 13 proteins could be divided into 4 clades based on the presence of the transmembrane region and the position of CFEM domain in the whole sequence. Six MaCFEM proteins with a signal peptide and without a transmembrane domain were considered candidate effector proteins. According to Phyre2 analysis, the *MaCFEM*88 and *MaCFEM*85 have the most homologous to Csa2 in *Candida albicans*. Subcellular localization analysis revealed that five effectors were located in the plasma membrane, while MaCFEM88 may locate in both plasma membrane and nucleus in the treated *Nicotiana benthamiana*. Expression pattern analysis showed that MaCFEM81, 85, 88, and 89 expression level was significantly higher in the sporulation stage compared to other growth stages. Furthermore, the yeast secretion assay showed that six candidate effectors were able to secrete out of the cell. All of the MaCFEMs couldn’t affect INF1-induced programmed cell death (PCD), but MaCFEM85 and 88 could trigger a slight hypersensitive response both when applied separately or in combination with INF1 in *N. benthamiana* leaves. These findings showed that six MaCFEM potential effectors with various structures and subcellular localizations in host cells might be used to illustrate the roles of MaCFEM proteins during *M. anisopliae*-plant interactions.

## 1. Introduction

Fungi in the order of Hypocreales (Ascomycota) are well known for their diverse lifestyles, including saprophytism, endogenous, and parasitism [1]. *Metarhizium* Sorokīn (Hypocreales, Clavicipitaceae) is one of the most well-studied and ubiquitous fungi, fulfilling roles that include as entomopathogens and facultative endophytes [2]. The recognition of a potential endophytic role was first elucidated by Hu and St. Leger 2002, where it was shown that the propagules of *M. anisopliae* (Metschn.) Sorokīn in the inner rhizosphere soil was four times higher than in the outer rhizosphere of cabbage roots [3]. Nearly a decade later, Elena et al. (2011) inoculated *M. anisopliae* conidia near the base of the shoot of tomato seedlings and demonstrated that *M. anisopliae* was also able to function as an endophyte [4]. Since then, several methods have been used to confirm the endosymbiotic link between *M. anisopliae* and plants, including fluorescence microscopy, tissue isolation, and isotopic labelling [5,6,7].

Plants utilize the complex community of rhizospheric microbes to maintain health and primary production [8]. These microbes vary in their ecological roles and provide benefits that include the stimulation of plant growth [9], competitive suppression of pathogens through secondary metabolites or spatial restriction [10], and increased resistance to biotic and abiotic stress [11].

When associated with the rhizosphere or beneficial root endophytes. *Metarhizium* was been shown to facilitate the transfer of nutrients to their plant hosts, including nitrogen [12,13], phosphorous [14], iron (Fe) [15,16]. Moreover, *Metarhizium* was also beneficial in mitigating high salt-induced oxidative stress conditions and improving the quantity and quality of crops, providing benefits such as resistance to salt stress [17]. *Metarhizium* preferentially interact with the plant rhizosphere when inoculated, resulting in a considerable reduction in disease incidence, development, and severity [18]. However, little research has been conducted on how *Metarhizium* influences the defensive response of plants and what effectors communicate information between *Metarhizium* and the plant.

Effectors are secreted microbial molecules that change plant processes and aid colonization. They are crucial to comprehending the complex interplay between plants and microorganisms. Hundreds of (virulence) microbe effector proteins are transported into the cytosol and apoplast of the host plant to modify plant defences and physiology by pathogens and beneficial microorganisms [19]. Common in fungal extracellular membrane proteins (CFEMs) have recently received considerable attention. These proteins have about 60 amino acid residues, including eight conserved cysteine residues [20]. According to systematic comparative genomic investigations, the CFEM domain is unique to fungi, and its origin goes back to the most recent common ancestor of Ascomycota and Basidiomycota [21]. The CFEM domain-containing fungal genes were mainly studied in plant pathogens, such as *Colletotrichum graminicola* (Ces.) G.W.Wilson [22], *Fusarium oxysporum* Schltdl. [23] and *Setosphaeria turcica* (Luttr.) K.J.Leonard & Suggs [24] and also reported in the animal pathogen *Candida albicans* (C.P.Robin) Berkhout [25]. CFEM proteins are linked with various functions of fungi, such as Pth11 in *Magnaporthe oryzae* B.C.Couch, which is associated with appressorium formation [26], Csa2 in *C. albicans* involved in the utilization of hemoglobin as an iron source [25], and CfemA-C in *Aspergillus fumigatus* for stabilization of the cell wall [27]. However, comprehensive identification and understanding the function of CFEM domain-containing proteins in *M. anisopliae* is lacking.

In order to understand the interaction between *M. anisopliae* and plants, we investigated six CFEM proteins associated with *M. anisopliae*, and using *Nicotiana benthamiana* Domin as the model plant and investigated subcellular localization, different transcription levels and diverse cell-death suppression activities.

## 2. Materials and Methods

### 2.1. Biomaterials and Their Culture Conditions

The *M. anisopliae* Ma9 strain was isolated and preserved in the laboratory. yeast YTK12 strain and pSuc2 vector were supplied by professor Wei Guo (Institute of Food Science and Technology, CAAS).

Ma9 was maintained on potato sucrose agar yeast extract medium (PSAY: 2% sucrose, 20% potato, 0.5% yeast extraction, 1.5% agar) and cultured at 26 °C for 8–10 days. Yeast YTK12 strain was routinely grown on yeast extract peptone dextrose medium (YPD: 1% yeast, 2% peptone, 2% glucose, 0.003% sulfate adenine and 2% agar) and was maintained at 30°C constant temperature incubator. For transient expression of Agrobacterium GV3101 strain (Tsingke Biotechnology Co., Ltd., Beijing, China) was cultured using LB medium (1% tryptone, 0.5% yeast extraction and 1% NaCl). *N. benthamiana* was grown in an artificial climate chamber under a 14:10 h L:D ratio and 27 °C and 25 °C, respectively. The *Escherichia coli* (Migula) Castellani and Chalmers strain DH5α (Tsingke Biotechnology Co., Ltd. Beijing, China) was used for plasmid amplification and cultivated in LB medium on a shaking incubator at 37 °C. In this study, different antibiotics are selected according to different vectors and strains. The antibiotics rifampin, kanamycin and ampicillin were added at 25, 50 and 50 µg/mL, respectively.

### 2.2. Bioinformatic Identification of CFEM-Containing Proteins in M. anisopliae

To identify CFEM candidate proteins, Pfam database (http://pfam.xfam.org/, accessed on 20 February 2021) was used to identify the number and amino acid sequence by typing *Metarhizium anisopliae* in the search box. The reference nucleic acid sequences of these CFEM proteins were obtained using the basic local alignment search tool tBLASTn. Specific primers were designed by DNAMAN for all of the sequences to obtain the CFEM gene sequence of *M. anisopliae* Ma9. All obtained proteins were examined for the presence of CFEM domains using the Pfam tool on the SMART website (http://smart.embl-heidelberg.de/, accessed on 20 February 2021), and sequences without CFEM domains were manually omitted. The signal peptide sequences of these proteins were analyzed using the SignalP 5.0 Server (https://services.healthtech.dtu.dk/service.php?SignalP-5.0, accessed on 20 February 2021).

### 2.3. Phylogenetic Analysis and Multiple Sequences Alignment

To identify conserved amino acids in the MaCFEM proteins, the CFEM domain sequences were extracted, and a multiple sequence alignment using ClustalW was performed. The alignment result was modified and drawn with Genedoc Software and analyzed using WebLogo (http://weblogo.berkeley.edu/logo.cgi, accessed on 20 February 2021).

Multiple protein sequence alignments of MaCFEM proteins were obtained by using ClustalW default settings. The neighbor-joining method was used to construct a phylogenetic tree with the MEGA X Program. Bootstrap analysis was conducted using the *p*-distance model with 1000 replications. Additionally, the domain architecture of the MaCFEM was drawn with EVOLVIEW (https://www.evolgenius.info/evolview-v2/#login, accessed on 20 February 2021) in this study. The bioinformatics on these structures, including the position of CFEM domain and transmembrane regions, was obtained from the SMART website’s bioinformation analysis. The position of the signal peptide was analyzed by SignalP 5.0 server.

### 2.4. Protein Model Analysis of Candidate MaCFEM Effectors

Proteins with an N-terminal signal peptide and no transmembrane domain were candidate effectors. Phyre2 (http://www.sbg.bio.ic.ac.uk/phyre2, accessed on 20 February 2021) was employed to generate predicted structures of these effectors using the amino acid sequences of the six candidates.

### 2.5. Expression Pattern of Candidate MaCFEM Effectors

For collection of *M. anisopliae* mycelium. Newly cultured sporangia were suspended in 0.05% Tween 80, and the concentration was adjusted to 10^7^/mL after counting with a hemocytometer. Sterilized cellophane was cut to fit into a 9 cm dia petri dish and placed on solidified PSAY media. Each dish was inoculated with 100 uL 10^7^/mL *M. anisopliae* conidia suspension. The mycelium on the cellophane surface was collected every 12 h after inoculation up to 96 h (12, 24, 36, 48, 60, 72, 84 and 96 h), with three dishes of mycelia collected at each time period. All samples were stored at −80 °C for subsequent experiments.

Total *M. anisopliae* RNA was extracted using TRIzol reagent (Invitrogen, Carlsbad, CA, USA) according to the manufacturer’s instructions, with the resulting RNA qualified and quantified using a NanoPhotometer^®^ (Implen, Münich, Germany). First-strand cDNA synthesis was performed using a 5× All-In-One RT Master Mix (Applied Biological Materials Inc., Canada) according to the manufacturer’s instructions.

qRT-PCR was performed by 2× SYBR Green qPCR Master Mix from US EVERBRIGHT^®^ INC. and detected according to the manufacturer’s instructions by the ABI QuantStudio 5 system. The internal reference gene primers used for normalizing each sample were listed in Supplementary Table S1. The gene try (involved in tryptophan biosynthesis) was used as a reference control [28]. Three technical replicates were performed for the targeted samples, with relative expression levels determined using the 2^−ΔΔCt^ method. All results are expressed as a mean ± standard error (SE), with *p* < 0.05 deemed significant following one-way ANOVA using Duncan’s multiple-range test (SPSS version 20.0, SPSS).

### 2.6. Yeast Secretion Assay

Signal peptides are responsible for guiding the transmembrane transfer of newly synthesized proteins through cytomembrane or subcellular organelle membranes. The yeast secretion system was previously described to verify the function of signal peptide. Yeast signal trap system based on the vector pSuc2 carried a truncated invertase gene (Suc2) lacking both the initiation Met amino acid and signal peptide [29] and was used to identify the secreted activity of MaCFEM effector. The tested signal peptide sequence in addition with six bp of the two following amino acids of MaCFEM81, 85, 87, 88, 89, and 90 were amplified by PCR using primers designed with DNAMAN software (Supplementary Table S1) in these candidate effectors. The signal peptide sequences were cloned into the EcoRI and XhoI sites of the yeast vector pSuc2 and connected to the sucrase gene Suc2, which lacks the initiation codon and signal peptide, to express the SP-SUC2 fusion protein. The six MaCFEMSP signal peptides, empty pSUC2 vector, and the Pr1JSP signal peptide were inserted into plasmid pSUC2 and transformed into yeast strain YTK12 using the lithium acetate method [30], then incubated at 30 °C in CMD-W (minus Trp) medium (0.67% yeast N base without amino acids, 0.075% W dropout supplement, 2% sucrose, 0.1% glucose, and 2% agar).The six MaCFEMSP signal peptides, the Pr1JSP signal peptide, and the empty pSUC2 vector were inserted into plasmid pSUC2 and transformed into yeast strain YTK12 using lithium acetate method incubated at 30 °C in CMD-W (minus Trp) medium (0.67% yeast N base without amino acids, 0.075% W dropout supplement, 2% sucrose, 0.1% glucose, and 2% agar).

Invertase secretion with the signal peptide of SP-SUC2 fusion was assayed by plating the colonies of the invertase-negative yeast strain YTK12. The pSUC2 and pSUC2::Pr1J were used as negative and positive controls, plated on YPRAA medium (1% yeast extract, 2% peptone, 2% raffinose, and 2 μg/mL antimicyn A) plates containing raffinose (lacking glucose), respectively. Moreover, the invertase enzymatic activity was verified by reducing TTC (2, 3, 5-triphenyltetrazolium chloride) to insoluble red-colored triphenylformazan. The yeast transformants were incubated in 5 mL sucrose medium for 24 h at 30 °C. The pellet was collected, washed, re-suspended in distilled sterile water, and incubated with 0.1% of the colorless dye TTC at 35 °C for 35 min. Then the invertase enzymatic activity was indicated by a colorimetric change 5 min after incubation at room temperature.

### 2.7. Subcellular Localization and Hypersensitive Response Assay

Subcellular localization and hypersensitive response assay of MaCFEM effector were conducted by agroinfiltration method. The whole cDNA sequence of MaCFEM81, 85, 87, 88, 89 and 90 was amplified by PCR with primers designed with DNAMAN software (Supplementary Table S1) and was cloned in *EcoR* I and *Sal* I sites of PYBA-1132. INF was an elicitin of *Phytophthora infestans* (Mont.) de Bary, usually as effector-mediated induction of programmed cell death in the host [31], and was used as the positive control in the *N. benthamiana* infiltration bioassy. The final recombinant vectors were transformed into *Agrobacterium tumefaciens* (Smith & Townsend) Conn GV3101 was cultured in LB (Luria-Bertani) medium with 25 mg L^−1^ rifampicin and 50 mg L^−1^ kanamycin. To infect *N. benthamiana*, the recombinant strains were washed three times with acetosyringone (AS) buffer (10 mmol L^−1^ MgCl_2_, 10 µmol L^−1^ (acetosyringone) AS, 10 mmol L^−1^ 2-(N-morpholino) ethanesulfonic acid (MES), pH5.6) and infiltrated into leaves of 4-week-old *N. benthamiana* at an OD_600_ of 0.5 using separate syringes without the needle. After being cultured for 30 h, the treated leaves were collected and detected under a Zeiss LSM980 confocal microscope (Carl Zeiss, Germany) for localization tests. For hypersensitive response assay, the strains expressing MaCFEM effector proteins were infiltrated into leaves 24 h before the infiltration of the strain carrying INF1. The disease symptoms in the *N. benthamiana* leaves were noted at 4–5 days post-inoculation (dpi) until INF1-induced cell.

## 3. Results

### 3.1. Bioinformatics Identification of CFEM Protein Repertoire in M. anisopliae

**Figure 1:**
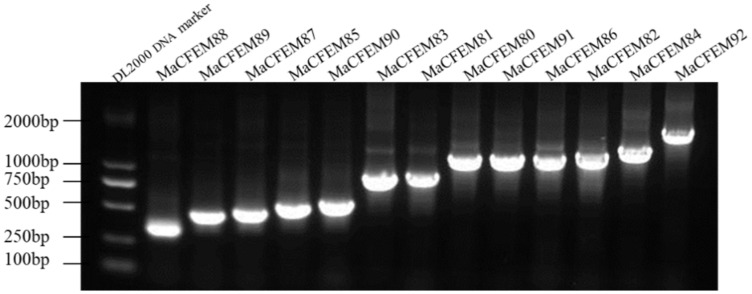
13 CFEM domain genes cloned in *Metarhizium anisopliae* Ma9 strain.

The 19 CFEM putative proteins of *M. anisopliae* were found using the Pfam database. Redundant genes were removed when tBLASTn was used to compare the genomes of *M. anisopliae*. 13 coding genes of the CFEM proteins were cloned and determined by sequencing and tBLASTn analysis (Figure 1). Further, CFEM domains in these proteins were verified by SMART analysis. The length of these proteins ranged from 105 (MaCFEM88) to 495 (MaCFEM84) amino acids, commonly containing one CFEM domain (except for MaCFEM86 with a double one). Their GenBank access ID and features are shown in Table 1.

**Figure 2:**
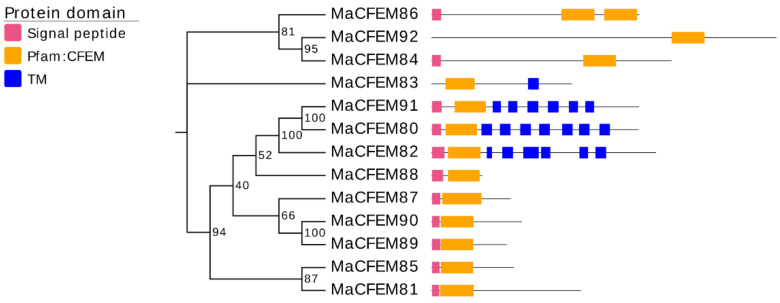
Phylogenetic analysis of CFEM proteins in *M. anisopliae.* A neighbour-joining phylogenetic tree was constructed based on the amino acid sequences of MaCFEM protein. The numbers at nodes represent the percentage of their occurrence in 1000 bootstrap replicates. The pink box represents the length and localization of the signal peptide, the orange box represents the CFEM domain, and the blue box represents the presence of a trans-membrane domain (TM).

A neighbor-joining phylogenetic tree was constructed based on the amino acid sequences of the MaCFEM proteins (Figure 2). These proteins could be divided into four clades. One clade included 3 proteins (MaCFEM84, 86, 92), in which the CFEM domain was near the C-terminus, specially MaCFEM86, with two CFEM domains connected in series. In other clades, the CFEM domain is positioned near the N-terminus. Each protein in the clade, including MaCFEM80, 82, and 91, had more than six transmembrane regions. The third clade included six proteins (MaCFEM81, 85, 87, 88, 89, 90) with signal peptides but without trans-membrane domains. The last clade had only one protein, MaCFEM83, which lacked a signal peptide but had a single transmembrane region.

**Figure 3:**
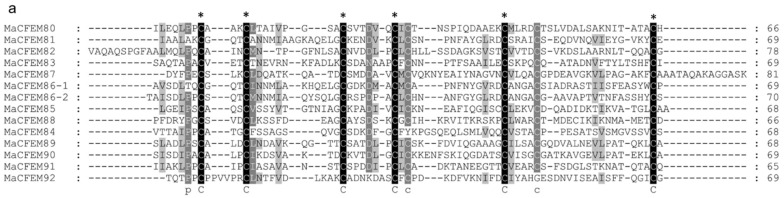

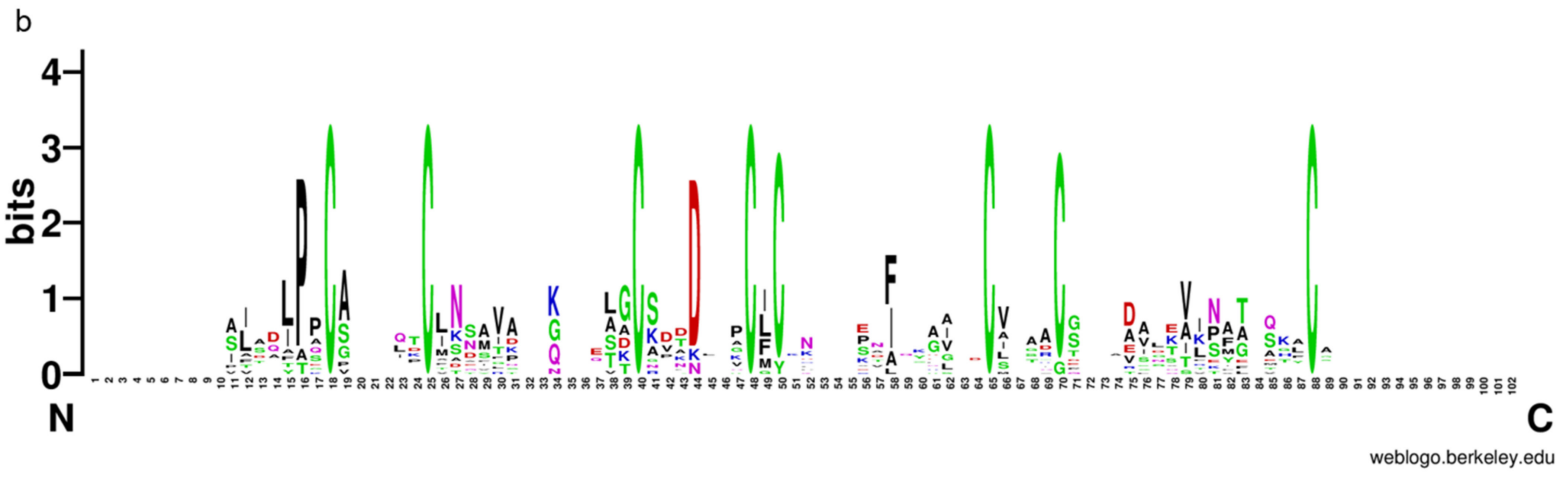
Multiple amino acid sequence alignment and WebLogo of CFEM domains from 13 proteins. (**a**) alignment was performed using ClustalW, and the result was modified with Gendoc. Conserved amino acid are highlighted in black and gray and marked with the corresponding letters. (**b**) For WebLogo, letters of prominent size indicate a relatively high degree of conservation.

To further analyze and elucidate the conserved amino acids in the CFEM domains, a multiple sequence alignment was performed ClustalW (Figure 3a). It was found that the CFEM domain was highly conserved in MaCFEMs evolution. Their length consists of about 70 aa (range between 65 and 81 aa) and usually contained eight characteristically spaced cysteines which form four disulfide bonds to help stabilize the whole structure of the domain. However, a few proteins, such as MaCFEM84 and 92, owned only seven conserved cysteines in the domains. WebLogo analysis also indicated that eight cysteines are highly conserved according to their letters prominent size (Figure 3b). The six CFEM proteins (MaCFEM81, 85, 87, 88, 89, and 90, consisting of about 300 aa), containing a signal peptide and no trans-membrane regions, could be candidate effectors and hence were selected for further analysis.

### 3.2. Helical-Basket Shape Detected in the Model Structure of CFEM Domain

**Figure 4:**
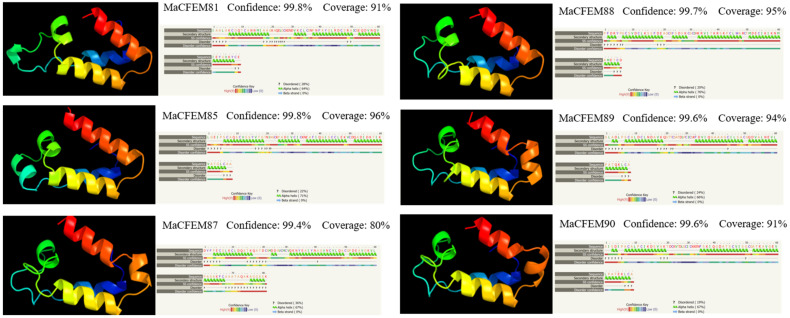
Analysis of structural models of MaCFEM effectors from the Phyre2 Server. The amino acid residues of these effectors have been modelled on the crystal structure of the CFEM domain in Csa2 from *C. albicans* with more than 90% confidence. The amino acid sequence of each effector is shown in the top line. The sequence on the next line shows residues colored according to a property-based scheme: yellow (A, S, T, G, P: small/polar), green (M, I, L, V: hydrophobic), red (K, R, E, N, D, H, Q: charged) and purple (W, Y, F, C: aromatic + cysteine). Green helices represent an α-helix, blue arrows indicate β-strands, and faint lines indicate coils.

The amino acid sequences of CFEM domains in six candidate effectors were uploaded in Phyre2 for modeling construction and protein homology analysis. The result showed that the amino acid sequences have high similarity to protein Csa2 (c4y7sC in *C. albicans*). Each CFEM domain was modeled with more than 97% confidence in Csa2. Protein Csa2 is the only identified three-dimensional (3D) crystal structure of CFEM protein [32]. The structure comprises 6 α-helices and 1 β-strand, which could form a helical-basket shape with an elongated N-terminal loop as a handle. Each testing model of the six CFEM domains also contains a helical-basket-like structure similar to Csa2. Four candidate effectors (MaCFEM81, 85, 89, 90) contain 4 α-helices, and the remaining two (MaCFEM87, 88) contain 5 α-helices (Figure 4).

### 3.3. Subcellular Localization Analysis of Six MaCFEM Effectors in N. benthamiana Leaves 

**Figure 5:**
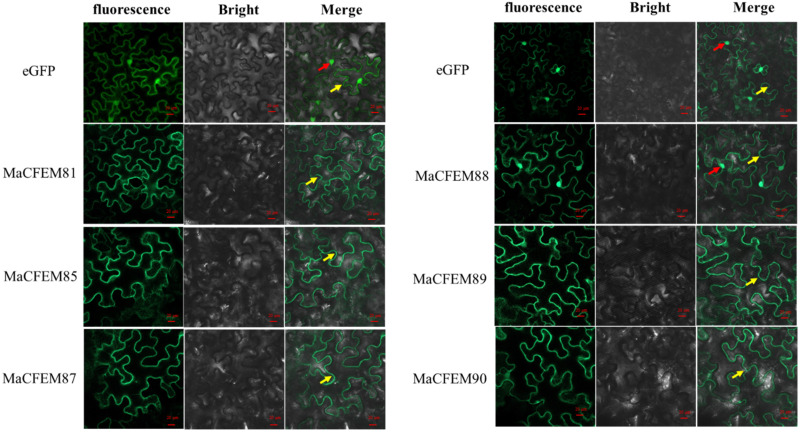
The subcellular location of MaCFEM effectors. The full length of MaCFEM effectors was fusion with eGFP proteins that cloned into PYBA1132 expression vector. The control vector was pYBA1132 carrying eGFP driven by the 35S promotor. By infiltrated 30 h, photos were taken under laser scanning confocal microscopy at eGFP (with 488 nm excitation and 507 nm emission), bright field and merge, respectively. Yellow arrow shows membrane boundaries (visiable as squiggly outlines) and red arrow highlighted green dots the nuclei.

The position of protein action is closely related to its function. Fungus-secreted effectors might connect with plants and spread inside plant cells, targeting various organelles and influencing protein activities, ultimately manipulating plant immunity [33,34]. The subcellular localization of six MaCFEM effectors was investigated in *N. benthamiana* leaves. The heterologous expression of MaCFEM full-length fusion with green fluorescent label gene *egfp* was conducted through the agroinfiltration method in *N. benthamiana*. The result showed in Figure 5 images that green fluorescence was detected in the plasma membrane (visiable as squiggly outlines) in the treatments of MaCFEM81, 85, 87, 89 and 90, while detected in both cell membrane and nucleus (visiable as bright spots) under MaCFEM88 role. Fluorescence may reflect where the MaCFEM proteins produce functions.

### 3.4. Expression Pattern of MaCFEM Effectors during the M. anisopliae Life Cycle

**Figure 6:**
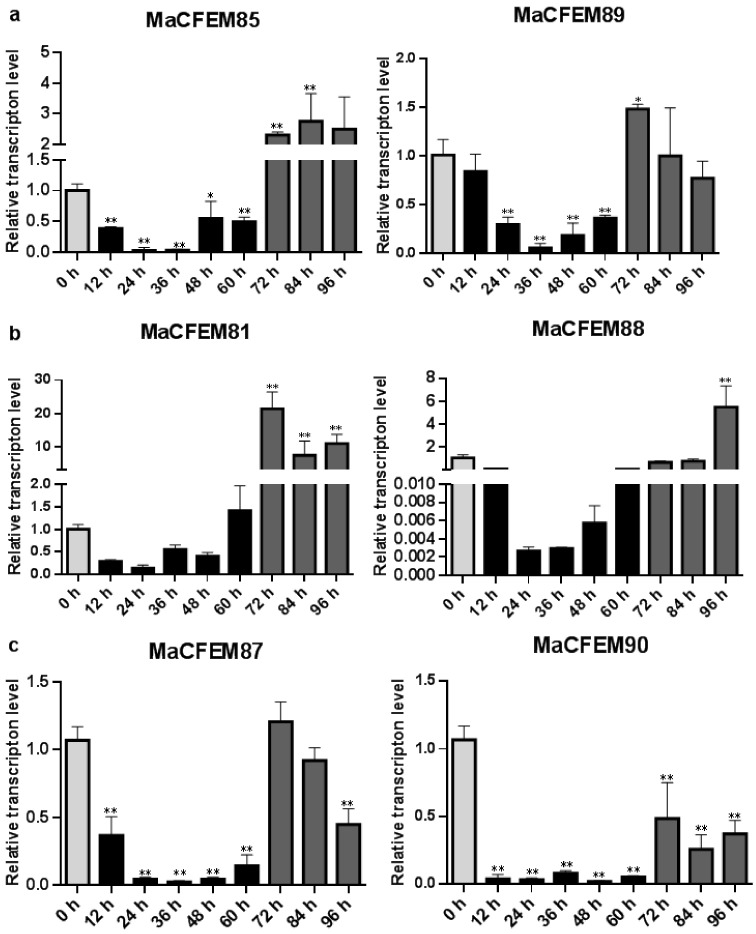
The expression patterns of MaCFEM effectors. qRT-PCR analysis of MaCFEM effectors expression in *M. anisopliae* life cycle (0–96 h) on PSA cultures. (**a**) Pattern I, containing MaCFEM85 and 89. (**b**) Pattern II, containing MaCFEM81 and 88. (**c**) Pattern III, containing MaCFEM87 and 90. Error bars show the standard deviation of three biological replications. Asterisks indicate statistically significant differences, as determined by one-way ANOVA using Duncan’s multiple-range test, * *p* < 0.05; ** *p* < 0.01.

The expression patterns of MaCFEM effectors were investigated over the *M. anisopliae* life cycle from conidial germination to hyphal development and conidiation, during the 96-h culture. Development may be roughly divided into several stages encompassing initial conidia (0 h), intracellular activation (0–12 h), germinating bud and growing hyphae (12–60 h), and conidiation formulation (60–96 h). Results showed that six MaCFEM effectors predominantly presented three expression patterns in various developmental stages compared to initial conidia (Figure 6). For MaCFEM85 and MaCFEM89, Pattern I showed their expression levels were significantly lower at germinating bud and growing hyphae stage compared to the initial conidia (MaCFEM85 at 24 and 36 h *p* < 0.01, MaCFEM89 at 24 and 36 h *p* < 0.01, and at 48 h, MaCFEM85 *p* = 0.0207, MaCFEM89 *p* < 0.01). The MaCFEM85 at 24 h and MaCFEM89 at 36 h showed the lowest expression level, only occupying 3.1% and 5.2% of initial conidia, respectively. They both showed considerably greater expression than the first conidia stage at 72 h, but higher expression observed for MaCFEM85 remained from 72 to 84 h (*p* < 0.01). However, no significant differences were observed in expression from 84 to 96 h and the initial conidia stage for MaCFEM89 (Figure 6a). Pattern II showed that the expression levels of MaCFEM81 and MaCFEM88 were only considerably greater in conidiation formulation than in the initial conidia stage, with no significant difference between the subsequent stages and the initial conidia. MaCFEM81 was expressed in all stages of sporulation (72, 84 and 96 h *p* < 0.01), with the highest expression at 72 h (*p* < 0.01). The expression was 21.3 times higher than the initial conidia stage. However, the MaCFEM88 expression was only considerably greater at 96 h (*p* < 0.01). It was 5.33 times higher than the initial conidia stage (Figure 6b). Pattern III indicated that MaCFEM87 and MaCFEM90 expression levels decreased in almost all stages compared to the first conidia stage (Figure 6c).

### 3.5. Functional Evaluation of the Signal Peptide of MaCFEM Effectors

**Figure 7:**
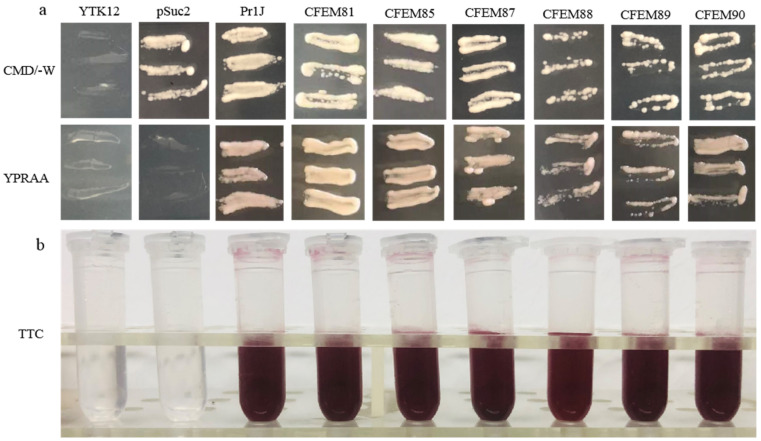
Functional evaluation of the signal peptide of MaCFEM six candidate effectors. (**a**) Secretion function of the signal peptide of candidate effectors was confirmed using a yeast secretion system. The function of signal peptides of six candidate effectors was identified using the *Saccharomyces cerevisiae* strain YTK12 containing the pSuc2 vector, Pr1J, MaCFEM SP. Yeast transformants were cultured on CMD\-W medium (on which the yeast grew well without invertase secretion), sucrose plates, and YPRAA medium (yeast only grew with active invertase secretion). (**b**) Invertase activity was detected by reduction of 2,3,5-triphenyltetrazolium chloride (TTC) to the insoluble, red colored 1,3,5-triphenylformazan (TPF). Transformed cells were collected in tubes to detect invertase activity. The negative control is the YTK12 strain carrying pSuc2 empty vector, and the positive control is the YTK12 strain carrying the signal peptide of Pr1J. CFEM81, 85, 87, 88, 89, and 90 represent MaCFEM81SP, MaCFEM85SP, MaCFEM87SP, MaCFEM88SP, MaCFEM89SP, and MaCFEM90SP, respectively.

The signal peptides (SP) of the six candidate effectors (MaCFEM81, 85, 87, 88, 89 and 90) and positive control (Pr1J, a secretory protein on the fungus *M. anisopliae*), were successfully introduced into pSuc2 and transformed into an invertase mutant yeast strain, YTK12. These recombinant strains were streaked onto CMD\-W (with sucrose, absence of tryptophan) medium and YPRAA medium (with raffinose as the only carbon source). The results showed that on CMD\-W medium, the constructed yeast strains successfully grew on the plate, with the exception of YTK12 (without pSuc2 vector). However, both YTK12 and pSuc2 were absent in the YRPAA medium. In contrast, others were grown commonly on the medium (Figure 7a). This shows that these recombinant strains could successfully secrete invertase.

The enzyme activity of secreted invertase was detected by reducing 2,3,5-triphenyltetrazolium chloride (TTC). The transformants containing Pr1J SP and MaCFEM SP turned the reaction reagents red. The strain containing pSuc2 empty vector and the yeast strain YTK12 did not, indicating that the signal peptide of Pr1J SP and MaCFEMs could mediate invertase secretion (Figure 7b). These results confirmed the signal prediction of the software SignalP 5.0 and proved that the putative N-terminal signal peptides of MaCFEMs possessed the activity to secrete the enzymes.

### 3.6. Hypersensitive Response Assay of MaCFEM Effectors

**Figure 8:**
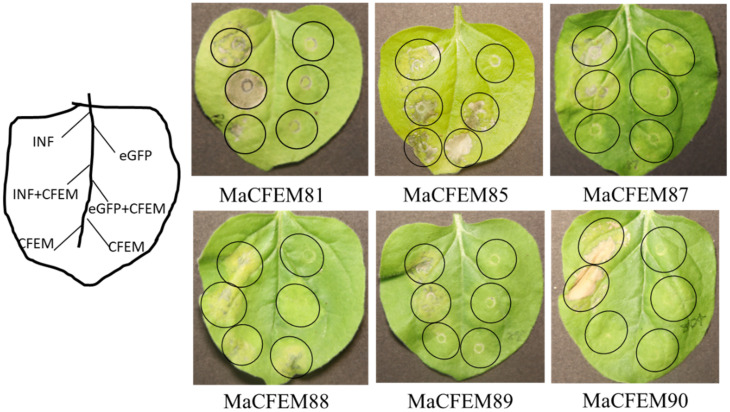
Transient expression analysis of six MaCFEM in suppression of INF-triggered cell death in *N. benthamiana*. *A. tumefaciens* carrying INF1, MaCFEM, empty vector (eGFP) or INF + MaCFEM, eGFP + MaCFEM, was infiltrated on tobacco leaf to detect the hypersensitive response, respectively. INF was used as a positive control, and empty vector (eGFP) used as the negative control. The results were recorded five days post-inoculation (dpi).

Fungal secreting effectors can mediate plant immune response to ensure a successful invasion. The effectiveness of CFEM domain effectors in activating or inhibiting plant immune responses has been reported [22,35]. INF1-induced programmed cell death (PCD) was used as a positive control to see if these MaCFEM effectors might inhibit or activate cell death in the non-host plant *N. benthamiana*. Via injecting MaCFEMs and positive control, the effect on PCD induced by positive control was observed. The results showed that CFEM81, 87, 89, and 90 neither induce nor suppress plant immune response. MaCFEM85 and MaCFEM88 could not suppress INF1-induced programmed cell death but could activate slight programmed cell death in *N. benthamiana* (Figure 8).

## 4. Discussion

The CFEM domain protein is considered a unique protein found only in fungi. Its origin links to the most recent common ancestors of Ascomycota and Basidiomycota [21], which means not all fungi contain this protein. The CFEM family protein has been extensively described in relation to plant pathogens, with the number and function of effectors also found to vary with species. In *C. graminicola*, 24 CgCFEM proteins were identified from the fungal genome. Five effectors such as CgCFEM6, 7, 8, 9, and 15 were deemed to suppress the BAX-induced programmed cell death (PCD) in *N. benthamiana* [22]. Similarly, 13 StCFEM proteins were identified in the genome of *S. turcica*, only one (StCFEM12) of the four effectors with different functions was found to suppress BAX/INF1-induced PCD, whereas the others (StCFEM3, 4, and 5) had no impact [24]. The function of the effectors in some fungi was rarely described in CFEM domain family proteins. *Fusarium graminearum* Schwabe was identified with 23 FgCFEM proteins from the genome [36]. From 12 different *F. oxysporum* formae species (f. sp), an average of 16 CFEM family member proteins were identified [23]. In this study, we retrieved 19 CFEM domain proteins in *Metarhizium* genus from Pfam database. Furthermore, 13 CFEM protein members were successfully cloned and identified from *M. anisopliae* strain Ma9 and all of them were sequenced (Figure 1). We hypothesized that the six potential CFEM proteins unsuccessfully cloned were distinct from *M. anisopliae* or the diversity of post-transcriptional gene translation. Nonetheless, since the reported sequences of sibling species may be a useful reference, it is still possible to examine CFEM proteins in related species with unknown genome sequences. Herein for the first time, the CFEM domain protein has been identified in the entomopathogenic and facultative endophytic fungus *M. anisopliae*.

Effectors are key initiators of fungus-plant molecular language communication. Microbes manipulated the plant immune to realize colonization by releasing effectors [37]. So far, most CFEM proteins were reported in pathogenic fungi, and the most of them as effectors contain the characteristics with signal peptides without trans-membrane domain. Effector proteins are inhibitory proteins of host immunity that assist pathogens in evading immunity and establishing a successful infection. However, not all CFEM effectors have immunosuppressive effects. For example, In *C. graminicola*, only five of the ten effectors have been shown to host immune suppression [22]. In the six effectors of *S. turcica*, only StCFEM12 could suppress the PCD of the host [24]. Recent documents reported an immune suppression mechanism of CFEM effector. Due to the conserved site of iron-binding aspartic acid residue (Asp) transformed into asparagine residue (Asn), two CFEM proteins of Verticillium dahlia, VdSCP76 and VdSCP77, mediated broad-spectrum inhibition of all immunological responses elicited by usual effectors [38]. Some CFEM effectors might trigger particular immunity in addition to immune suppression. The CFEM effector candidate PTTG 08198 enhanced cell death and promoted the accumulation of reactive oxygen species (ROS) in the wheat leaf rust fungus *Puccinia triticina* [39]. In *Botrytis cinerea*, BcCFEM1 protein could activate plant immune response by inducing chlorosis in *N. benthamiana* [35]. According to our findings, six MaCFEM effectors produced different immunological responses in *N. benthamiana*. All six MaCFEM effectors could not suppress INF-induced programmed cell death, while MaCFEM85 and MaCFEM88 could slightly induce PCD alone (Figure 8). Similar results also showed in *Lasiodiplodia theobromae*, two CFEM proteins, LtCFEM1 and LtCFEM14, were shown to cause a local yellowish phenotype and cell death lesion in *N. benthamiana* leaves, but the other six LtCFEM proteins did not show any signs of PCD in leaves and did not activate the plant immune response [40]. The mechanism of stimulating the host immune system remains unclear. Further study is needed to fully understand the CFEM proteins’ complex actions.

In addition, CFEM protein is involved in the formation and stability of biofilms and cell walls and has a key role in penetrating the host [41,42]. Biofilms are clusters of biomacromolecules such as proteins, polysaccharides, DNA, RNA, peptidoglycans, lipids, and phospholipids that enhance fungal adhesion to the host and overcome host defenses and immunity [43].

According to previous research, there are two types of CFEM protein that keep cell wall and biofilm stability. One type contains a cross membrane domain and has a close binding relationship with cell wall. This type was illustrated by the CFEM protein Pth11 in *M. oryzae*. Pth11 is a G-protein-coupled receptor-like protein with seven transmembrane regions. After the knockout of Pth11 gene, the appressorium on the hydrophobic surface was weakened and failed to penetrate the leaves of susceptible rice. This shows that Pth11 is linked to cell wall integrity and hydrophobicity [41]. In *B. Cinerea*, the CFEM protein Bcin07g03260 contains only one transmembrane domain, which differs from the G-protein-coupled receptor. When the Bcin07g03260 gene was knockout, the fungus would significantly reduce spore germination and bud tube elongation resulting decreased infection rate on leaves [44]. In this study, three CFEM proteins from *M. anisopliae* have multiple transmembrane regions (CFEM82 and CFEM91 with six transmembrane regions, CFEM80 with seven transmembrane regions), and one (CFEM83) has a single transmembrane region (Figure 2). The role of these proteins in the cell wall and biofilm stability is still unknown in *M. anisopliae*.

The GPI-anchored structure is another type of CFEM protein that is involved in cell wall stability. In *A. fumigatus*, three CFEM proteins with a GPI-anchored structure were discovered. When single, two or three genes of the three proteins were knocked out respectively, the mutants increased the sensitivity in fungal cell wall inhibitors Congo red (CR) and calcium fluorescent protein (CFW), but did not affect spore germination and growth [27]. In *B. cinerea*, the BcCFEM1, which also contains GPI-anchor structure, the absence of BcCFEM1 were inhibited growth in various stress media (CR, CFW, SDS, NaCl etc.), but did not affect spore germination, growth rate, colony morphology and sclerotia formation [35]. When the GPI-anchor contained in gene SCR76 was knocked out in *Verticillium dahlia*, the surface of the spores became wrinkled and sticky, the outer wall of the spores was uneven, and the organelles were not well characterized [45]. Otherwise, deletion of three CFEM-encoding genes (Rbt5/Rbt51/Csa1) enhances sensitivity to cell-wall damaging agents and reduces the ability to produce an autologous biofilm in the zoonotic fungus *C. albicans* [46]. The above examples suggested that CFEM proteins have a variety of functions, including maintain cell wall stabilization. Proteins lacking a transmembrane domain may also impact the integrity of fungal cell walls and biofilms. Among the six identified MaCFEM effectors in this experiment, five were predicted to contain GPI-anchor points except for MaCFEM88 (Supplementary Table S2). They were predicted by a GPI-anchor predictor PredGPI (http://gpcr.biocomp.unibo.it/predgpi/pred.htm, accessed on 20 February 2021) but failed to determine whether these proteins were related to the physiological characteristics such as spore germination, growth, sporulation or cell wall stability. Among the six effectors, only MaCFEM88 lacked the GPI anchor site, and its subcellular localization was in both cell membrane and nucleus which different from that of the other five effectors (only in cell membrane). This implies a correlation between GPI and subcellular localization. Some proteins could be modified C-terminal with glycosylphosphatidylinositol (GPI), and then anchored on the cell surface with the lipid component. The GPI anchor plays an important role in cell membrane localization. In the case of *Arabidopsis thaliana* (L.) Heynh., deletion of the GPI anchor site of the Arabinoglactan protein gene AtFLA3 resulted in the eGFPm-AtFLA3△GPI protein originally located in the cell membrane became confused and shift to the extracellular matrix in an unstable form [47]. It suggests that the GPI site facilitate protein anchoring in the plasma membrane. This may be why MaCFEM88 localization is different from the other five effectors, due to lacking the GPI anchor site. In addition, the diversity of subcellular localization in a protein family is universal. For example, members of the glycine-rich protein superfamily are diverse in subcellular localization and function differently under different localization conditions [48]. *A. thaliana* AtGRP2 expressed in both cytoplasm and nucleus, which can help the plant resist cold, salt and osmotic stress, while also having a role in RNA chaperone activity [49,50]. This suggests that the MaCFEM88 subcellular localization may indicate some specific functions different from the other five MaCFEM effectors, although this aspect needs to explored and verified.

## 5. Conclusions

In this study, we successfully cloned and identified 13 MaCFEM domain proteins in *M. anisopliae*. Six of them were considered candidate effectors due to the presence of signal peptides and lack of transmembrane domains. The five proteins (MaCFEM81, 85, 87, 89, 90) were functionally located in the plant plasma membrane, while MaCFEM88 seems located both in nucleus and plasma membrane. The MaCFEM81, 85, 88, and 89 were significantly upregulated in the conidiation stage. Although a minor hypersensitive response (HR) reaction was identified in MaCFEM85 and MaCFEM88 induction, none of the candidate effectors effectively reduced the HR, INF1-induced program cell death in *N. benthamiana*.

## Figures and Tables

**Table 1:** Identified features of the *M. anisopliae* CFEM proteins.

Protein ID	Name	Family	Amino Acids (aa)	Molecular Weight (Da)	Position of CFEM Domain (aa)	E-Value
OL477622	MaCFEM80	CFEM	427	47,132	29–94	3.20 × 10^−13^
OL477623	MaCFEM81	CFEM	308	28,905	18–87	1.30 × 10^−11^
OL477624	MaCFEM82	CFEM	463	51,102	34–101	8.10 × 10^−11^
OL477626	MaCFEM87	CFEM	163	15,907	22–103	1.20 × 10^−8^
OL477627	MaCFEM86	CFEM	429	44,923	268–336/356–424	2.5 × 10^−10^/5.2 × 10^−13^
MZ682609	MaCFEM85	CFEM	170	16,364	19–86	1.30 × 10^−13^
OL477629	MaCFEM84	CFEM	495	50,794	313–380	1.40 × 10^−7^
OL477630	MaCFEM89	CFEM	155	14,983	19–86	1.20 × 10^−13^
OL477631	MaCFEM90	CFEM	186	18,076	19–87	1.10 × 10^−12^
OL477632	MaCFEM91	CFEM	428	46,326	48–112	2.40 × 10^−13^
OL477633	MaCFEM92	CFEM	711	74,011	495–563	2.30 × 10^−5^
OL477628	MaCFEM88	CFEM	105	11,453	34–99	1.20 × 10^−9^
OL477625	MaCFEM83	CFEM	290	30,111	21–89	3.10 × 10^−7^

## Data Availability

The data presented in this study are available in this article and its Supplementary Materials.

## Supplementary Materials

The following supporting information can be downloaded at: https://www.mdpi.com/article/10.3390/jof8070661/s1, Table S1: The primers used in this article; Table S2: The results of six MaCFEM effectors GPI-anchor sites predicted by PredGPI.

## References

[1] Zhang W., Zhang X., Li K., Wang C., Cai L., Zhuang W., Xiang M., Liu X. (2018). Introgression and gene family contraction drive the evolution of lifestyle and host shifts of hypocrealean fungi. Mycology.

[2] Wang J.B., St. Leger R.J., Wang C., Lovett B., St. Leger R.J. (2016). Chapter Three—Advances in Genomics of Entomopathogenic Fungi. Advances in Genetics.

[3] Hu G., St. Leger R.J. (2002). Field studies using a recombinant mycoinsecticide (*Metarhizium anisopliae*) reveal that it is rhizosphere competent. Appl. Environ. Microbiol..

[4] Campos M.L., Kang J.H. (2014). Howe GA: Jasmonate-triggered plant immunity. J. Chem. Ecol..

[5] Sasan R.K., Bidochka M.J. (2012). The insect-pathogenic fungus *Metarhizium robertsii* (Clavicipitaceae) is also an endophyte that stimulates plant root development. Am. J. Bot..

[6] Mejia L.C., Herre E.A., Sparks J.P., Winter K., Garcia M.N., Van Bael S.A., Stitt J., Shi Z., Zhang Y., Guiltinan M.J. (2014). Pervasive effects of a dominant foliar endophytic fungus on host genetic and phenotypic expression in a tropical tree. Front. Microbiol..

[7] Behie S.W., Moreira C.C., Sementchoukova I., Barelli L., Zelisko P.M., Bidochka M.J. (2017). Carbon translocation from a plant to an insect-pathogenic endophytic fungus. Nat. Commun..

[8] Jacoby R., Peukert M., Succurro A., Koprivova A., Kopriva S. (2017). The role of soil microorganisms in plant mineral nutrition-current knowledge and future directions. Front. Plant Sci..

[9] Dennis P.G., Miller A.J., Hirsch P.R. (2010). Are root exudates more important than other sources of rhizodeposits in structuring rhizosphere bacterial communities?. FEMS Microbiol. Ecol..

[10] Tan S., Gu Y., Yang C., Dong Y., Mei X., Shen Q., Xu Y. (2015). *Bacillus amyloliquefaciens* T-5 may prevent Ralstonia solanacearum infection through competitive exclusion. Biol. Fertil. Soils.

[11] Saijo Y., Loo E.P. (2020). Plant immunity in signal integration between biotic and abiotic stress responses. New Phytol..

[12] Behie S.W., Zelisko P.M., Bidochka M.J. (2012). Endophytic insect-parasitic fungi translocate nitrogen directly from insects to plants. Science.

[13] Behie S.W., Bidochka M.J. (2014). Ubiquity of insect-derived nitrogen transfer to plants by endophytic insect-pathogenic fungi: An additional branch of the soil nitrogen cycle. Appl. Environ. Microbiol..

[14] Krell V., Unger S., Jakobs-Schoenwandt D., Patel A.V. (2018). Importance of phosphorus supply through endophytic *Metarhizium brunneum* for root: Shoot allocation and root architecture in potato plants. Plant Soil.

[15] Sánchez-Rodríguez A.R., Barrón V., Del Campillo M.C., Quesada-Moraga E. (2016). The entomopathogenic fungus *Metarhizium brunneum*: A tool for alleviating Fe chlorosis. Plant Soil.

[16] Raya–Díaz S., Quesada–Moraga E., Barrón V., del Campillo M.C., Sánchez–Rodríguez A.R. (2017). Redefining the dose of the entomopathogenic fungus *Metarhizium brunneum* (Ascomycota, Hypocreales) to increase Fe bioavailability and promote plant growth in calcareous and sandy soils. Plant Soil.

[17] Khan A.L., Hamayun M., Khan S.A., Kang S.M., Shinwari Z.K., Kamran M., Rehman S.U., Kim J.G., Lee I.J. (2012). Pure culture of *Metarhizium anisopliae* LHL07 reprograms soybean to higher growth and mitigates salt stress. World J. Microbiol. Biotechnol..

[18] Jaber L.R. (2018). Seed inoculation with endophytic fungal entomopathogens promotes plant growth and reduces crown and root rot (CRR) caused by *Fusarium culmorum* in wheat. Planta.

[19] Jones J.D.G., Dangl J.L. (2006). The plant immune system. Nature.

[20] Bujalowski W. (2003). Expanding the physiological role of the hexameric DnaB helicase. Trends Biochem. Sci..

[21] Zhang Z.N., Wu Q.Y., Zhang G.Z., Zhu Y.Y., Murphy R.W., Liu Z., Zou C.G. (2015). Systematic analyses reveal uniqueness and origin of the CFEM domain in fungi. Sci. Rep..

[22] Gong A.D., Jing Z.Y., Zhang K., Tan Q.D., Wang G.L., Liu W.D. (2020). Bioinformatic analysis and functional characterization of the CFEM proteins in maize anthracnose fungus *Colletotrichum graminicola*. J. Integr. Agric..

[23] Ling J., Zeng F., Cao Y., Zhang J., Chen G., Mao Z., Yang Y., Xie B. (2015). Identification of a class of CFEM proteins containing a new conserved motif in *Fusarium oxysporum*. Physiol. Mol. Plant Pathol..

[24] Wang J.X., Long F., Zhu H., Zhang Y., Wu J.Y., Shen S., Dong J.G., Hao Z.M. (2021). Bioinformatic analysis and functional characterization of CFEM proteins in *Setosphaeria turcica*. J. Integr. Agric..

[25] Okamoto-Shibayama K., Kikuchi Y., Kokubu E., Sato Y., Ishihara K. (2014). Csa2, a member of the Rbt5 protein family, is involved in the utilization of iron from human hemoglobin during *Candida albicans* hyphal growth. FEMS Yeast Res..

[26] Kou Y., Tan Y.H., Ramanujam R., Naqvi N.I. (2017). Structure-function analyses of the Pth11 receptor reveal an important role for CFEM motif and redox regulation in rice blast. New Phytol..

[27] Vaknin Y., Shadkchan Y., Levdansky E., Morozov M., Romano J., Osherov N. (2014). The three *Aspergillus fumigatus* CFEM-domain GPI-anchored proteins (CfmA-C) affect cell-wall stability but do not play a role in fungal virulence. Fungal. Genet. Biol..

[28] Fang W., Bidochka M.J. (2006). Expression of genes involved in germination, conidiogenesis and pathogenesis in *Metarhizium anisopliae* using quantitative real-time RT-PCR. Mycol. Res..

[29] Liu S.F., Wang G.J., Nong X.Q., Liu B., Wang M.M., Li S.L., Cao G.C., Zhang Z.H. (2017). Entomopathogen *Metarhizium anisopliae* promotes the early development of peanut root. Plant Prot. Sci..

[30] Hao K., Wang F., Nong X., McNeill M.R., Liu S., Wang G., Cao G., Zhang Z.H. (2017). Response of peanut *Arachis hypogaea* roots to the presence of beneficial and pathogenic fungi by transcriptome analysis. Sci. Rep..

[31] Ownley B.H., Gwinn K.D., Vega F.E. (2009). Endophytic fungal entomopathogens with activity against plant pathogens: Ecology and evolution. BioControl.

[32] Nasser L., Weissman Z., Pinsky M., Amartely H., Dvir H., Kornitzer D. (2016). Structural basis of haem-iron acquisition by fungal pathogens. Nat. Microbiol..

[33] Aung K., Xin X., Mecey C., He S.Y. (2017). Subcellular Localization of *Pseudomonas syringae* pv. tomato effector proteins in plants. Methods Mol. Biol..

[34] Pradhan A., Ghosh S., Sahoo D., Jha G. (2021). Fungal effectors, the double edge sword of phytopathogens. Curr Genet..

[35] Zhu W., Wei W., Wu Y., Zhou Y., Peng F., Zhang S., Chen P., Xu X. (2017). BcCFEM1, a CFEM domain-containing protein with putative GPI-Anchored site, is involved in pathogenicity, conidial production, and stress tolerance in *Botrytis cinerea*. Front. Microbiol..

[36] Chen L., Wang H., Yang J., Yang X., Zhang M., Zhao Z., Fan Y., Wang C., Wang J. (2021). Bioinformatics and transcriptome analysis of CFEM proteins in *Fusarium graminearum*. J. Fungi.

[37] Lucke M., Correa M.G., Levy A. (2020). The role of secretion systems, effectors, and secondary metabolites of beneficial rhizobacteria in interactions with plants and microbes. Front. Plant Sci..

[38] Wang D., Zhang D.D., Song J., Li J.J., Wang J., Li R., Klosterman S.J., Kong Z.Q., Lin F.Z., Dai X.F. (2022). *Verticillium dahliae* CFEM proteins manipulate host immunity and differentially contribute to virulence. BMC Biol..

[39] Zhao S., Shang X., Bi W., Yu X., Liu D., Kang Z., Wang X., Wang X. (2020). Genome-wide identification of effector candidates with conserved motifs from the wheat leaf rust fungus *Puccinia triticina*. Front. Microbiol..

[40] Peng J., Wu L., Zhang W., Zhang Q., Xing Q., Wang X., Li X., Yan J. (2021). Systemic identification and functional characterization of common in fungal extracellular membrane proteins in *Lasiodiplodia theobromae*. Front. Plant Sci..

[41] Sabnam N., Roy Barman S. (2017). WISH, a novel CFEM GPCR is indispensable for surface sensing, asexual and pathogenic differentiation in rice blast fungus. Fungal Genet. Biol..

[42] Okamoto-Shibayama K., Kikuchi Y., Kokubu E., Ishihara K. (2017). Possible involvement of surface antigen protein 2 in the morphological transition and biofilm formation of *Candida albicans*. Medi Mycol. J..

[43] Saranathan R., Pagal S., Prashanth K., Mandal S., Paul D. (2019). Biofilm and Antibiotic Resistance in *Acinetobacter baumannii*. Bacterial Adaptation to Co-Resistance.

[44] Arya G.C., Srivastava D.A., Pandaranayaka E.P.J., Manasherova E., Prusky D.B., Elad Y., Frenkel O., Dvir H., Harel A. (2020). Characterization of the Role of a Non-GPCR membrane-bound cfem protein in the pathogenicity and germination of *Botrytis cinerea*. Microorganisms.

[45] Wang D. (2020). Functional Analysis of CFEM Domain-Containing Small Secreted Cysteine-Rich Proteins in *Verticillium dahliae*. Ph.D. Thesis.

[46] Srivastava V.K., Suneetha K.J., Kaur R. (2014). A systematic analysis reveals an essential role for high-affinity iron uptake system, haemolysin and CFEM domain-containing protein in iron homoeostasis and virulence in *Candida glabrata*. Biochem. J..

[47] Li J. (2010). Studies on the Biological Function of Arabinoglactan Protein Gene AtFLA3 in *Arabidopsis thaliana*. Ph.D. Thesis.

[48] Mangeon A., Junqueira R.M., Sachetto-Martins G. (2010). Functional diversity of the plant glycine-rich proteins superfamily. Plant Signal Behav..

[49] Park S.J., Kwak K.J., Oh T.R., Kim Y.O., Kang H. (2009). Cold shock domain proteins affect seed germination and growth of *Arabidopsis thaliana* under abiotic stress conditions. Plant Cell Physiol..

[50] Sasaki K., Kim M.H., Imai R. (2007). Arabidopsis COLD SHOCK DOMAIN PROTEIN2 is a RNA chaperone that is regulated by cold and developmental signals. Biochem. Biophys. Res. Commun..

