# Supplementary material for: Bioinformatics Analysis and Functional Characterization of the CFEM Proteins of Metarhizium anisopliae
Source: J Fungi (Basel). 2022 Jun 24;8(7):661. doi: 10.3390/jof8070661 (PMC9318983; doi:10.3390/jof8070661)
Supplement: Supplementary file 1 [file jof-08-00661-s001.zip › Supplement Table S1.pdf]

Gene cloning primers

CFEM80-F	ATGCGGTTCCCCATCAGC
CFEM80-R	TCACTGTTTGGCCTCGGACT
CFEM81-F	ATGAAGTCGGCTGCCTTC
CFEM81-R	TTAGATGAGGAGAGCGGC
CFEM82-F	ATGCGGGTTCCTTTTCAAG
CFEM82-R	CTATCCCTCTGCATGCTTTTGC
CFEM83-F	ATGGGTCGACTTTTACCGCT
CFEM83-R	TTACACCAAGGTCCTAGGCT
CFEM84-F	ATGCGGCCAT TGGTTCTCAC
CFEM84-R	CTACAGAGCC AGAGCGAG
CFEM85-F	ATGCGATCCTCATTCGTCACCTTG
CFEM85-R	TTAGAGAGCGGCAACGATGG
CFEM86-F	ATGATGCCTCTGTCCATC
CFEM86-R	TTATGGTCCCGGCGTAG
CFEM87-F	ATGAAGTCAACTCTGC
CFEM87-R	CTACAGGGCAGCCAACAGCC CG
CFEM88-F	ATGAAGCTTACCGCTGTTCTCG
CFEM88-R	TCAAAGTCCCTTCTCGATCCTG
CFEM89-F	ATGAAGTTCCTCGCCATTGCATC
CFEM89-R	TCACAGAGCCAACGCCGCG
CFEM90-F	ATGAAGTTCACTGCTGTCGCTGC
CFEM90-R	TTAGAGAGCCAGGGCACCGAT
CFEM91-F	ATGCGATTGCCATTTTGG
CFEM91-R	TCAACCCCCTTCCAGGT
CFEM92-F	ATGAAGTCTGCTCTTTCTGT TG
CFEM92-R	TTACAGAGCGGCGACGAAAG

qPCR primers

Try -F	TTGCAATGCATGTTTGATGTC
Try -R	CAAAGAGTGGTATCGAGTTAC
q CFEM81-F	GCAGCGAACAAGATGTCAA
q CFEM81-R	ACCGCCAGTAGCACTATGAG
q CFEM85-F	CAGCTCGGCGAAATCCCCTCAT
q CFEM85-R	TGGGAGTGTCGACGCCGCTG
q CFEM87-F	CCCGAGTGCTCTCTGAAGTGTC
q CFEM87-R	TGAGCAGTGGCAGCAGCACAG
q CFEM88-F	CCGCTGTTCTCGTCGCCATCTG
q CFEM88-R	CCCTCTTGTGGATGCAGCCGC
q CFEM89-F	GCCTCGCCGACCTGCCCAG
q CFEM89-R	GACGTGCCGGTGTTGCTGCC
q CFEM90-F	GTTCACTGCTGTCGCTGCTATC
q CFEM90-R	CGCAACTGGTAGCAGCACTCTGG

signal peptide assay primers

pSuc- CFEM81-F	GGAATTTTAATTAAGAATTCATGAAGTCGGCTGCC
pSuc- CFEM81-R	CTATAGGGAGAACCTCGAGGCACTTAGCGAGGGC
pSuc- CFEM85-F	GGAATTTTAATTAAGAATTCATGCGATCCTCATTC
pSuc- CFEM85-R	CTATAGGGAGAACCTCGAGCTGCTGTGCGGCTGC
pSuc- CFEM87-F	GGAATTTTAATTAAGAATTCATGAAGTCAACTCTGC
pSuc- CFEM87-R	CTATAGGGAGAACCTCGAGTCTGTTCTGCGCGAC
pSuc- CFEM88-F	GGAATTTTAATTAAGAATTCATGAAGCTTACCGCTG
pSuc- CFEM88-R	CTATAGGGAGAACCTCGAGGACGGCGGCGGAGGT
pSuc- CFEM89-F	GGAATTTTAATTAAGAATTCATGAAGTTCCTCGCC
pSuc- CFEM89-R	CTATAGGGAGAACCTCGAGCAGGTCGGCGAGGCT
pSuc- CFEM90-F	GGAATTTTAATTAAGAATTCATGAAGTTCACTGCT
pSuc- CFEM90-R	CTATAGGGAGAACCTCGAGGCTCTGAGCCGAGAC

HR assay primers

PYBA-CFEM81-F	caATTGGAGCTCCACCGCATGAAGTCGGCTGCCTT
PYBA-CFEM81-R	GGTACCGGGCCCCCCCTCGATGAGGAGAGCGGCAA
PYBA-CFEM85-F	caATTGGAGCTCCACCGCATGCGATCCTCATTCGTC
PYBA-CFEM85-R	GGTACCGGGCCCCCCCTCGAGAGCGGCAACGATGGCC
PYBA-CFEM87-F	caATTGGAGCTCCACCGCATGAAGTCAACTCTGCTCCTCTT
PYBA-CFEM87-R	GGTACCGGGCCCCCCCTCCAGGGCAGCCAACAGCCC
PYBA-CFEM88-F	caATTGGAGCTCCACCGCATGAAGCTTACCGCTGTTCT
PYBA-CFEM88-R	GGTACCGGGCCCCCCCTCAAGTCCCTTCTCGATCCTG
PYBA-CFEM89-F	caATTGGAGCTCCACCGCATGAAGTTCCTCGCCATTGC
PYBA-CFEM89-R	GGTACCGGGCCCCCCCTCCAGAGCCAACGCCGCGAA
PYBA-CFEM90-F	caATTGGAGCTCCACCGCATGAAGTTCACTGCTGTCGC
PYBA-CFEM90-R	GGTACCGGGCCCCCCCTCGAGAGCCAGGGCACCGAT
