# Supplementary material for: Bioinformatics Analysis and Functional Characterization of the CFEM Proteins of Metarhizium anisopliae
Source: J Fungi (Basel). 2022 Jun 24;8(7):661. doi: 10.3390/jof8070661 (PMC9318983; doi:10.3390/jof8070661)
Supplement: Supplementary file 1 [file jof-08-00661-s001.zip › Supplement Table S2.pdf]

Suplementary Table S2: GPI-anchor site predict of MaCFEM candidate effectors

protein name	omega-site position	Specificity	GPI-anchored
MaCFEM81	277	100.00%	yes
MaCFEM85	147	99.90%	yes
MaCFEM87	138	99.90%	yes
MaCFEM88	85	95.70%	no
MaCFEM89	134	100.00%	yes
MaCFEM90	165	99.90%	yes
